# Usefulness of avulsed fingertip skin for reconstruction after digital amputation

**DOI:** 10.1080/23320885.2020.1719843

**Published:** 2020-01-31

**Authors:** Atsuyoshi Osada, Hajime Matsumine, Wataru Kamei, Hiroyuki Sakurai

**Affiliations:** a Department of Plastic and Reconstructive Surgery, Tokyo Women’s Medical University, Tokyo, Japan

**Keywords:** Finger amputation, skin graft, donor, injury, cryopreserve, reconstruction

## Abstract

We present four cases of fingertip amputation treated with local flap where the avulsed fingertip skin could be effectively utilized for these donor sites. The avulsed finger skin may be utilized even when replantation is not possible. This approach may serve as a new treatment option after fingertip amputation.

## Introduction

Fingertip avulsion is a condition encountered frequently during injury treatment, with replantation being the surgical treatment of choice. However, in cases where replantation is difficult owing to the avulsion level, extent of injury or contamination, or facility environments, planning a reconstruction surgery tailored to individual cases is important. Given the diverse forms of avulsion, standardizing the reconstruction method is difficult. Numerous operative procedures for reconstruction using a local flap, free flap, etc. have been reported. If closure with a skin flap is selected, skin grafting is sometimes needed to close the wound of the flap donor site. In such cases, the operation cannot be completed within a single operative field, and sacrificing of the donor site cannot be avoided. Meanwhile, when dealing with avulsed fingertip cases, the avulsed finger not indicated for replantation is mostly discarded. No report on the utilization of such skin as a graft for the fingertip reconstruction donor site has been published. We have attempted to utilize the avulsed fingertip skin as a graft to fill the donor site needed for the local flap procedure of primary or secondary reconstructive surgery and have obtained favorable outcome. This attempt is reported in this paper, with reference to the literature.

## Operative procedure

This operative procedure was applied to cases where primary or secondary reconstruction with a local flap was planned after fingertip avulsion because of difficulty in replantation with microscopic vascular anastomosis because the avulsed finger was intensely crushed, distal phalanx exposure, and need of reconstruction of large volume finger pad. First, fingertip reconstruction by various local flap procedures was performed. The skin defect created in the donor site or the vascular pedicle was filled with a graft prepared by removing the bone/subcutaneous tissue of the avulsed finger and processing it into a full-thickness skin graft. If the avulsed skin contained nail matrix, its resection was needed. Only bandage dressing was used to fix the skin graft. The fixation was continued for 1 week. Tucking was not done. Finger movement was permitted 1–2 weeks postoperatively, depending on the status of flap/graft survival. For secondary reconstruction, the avulsed finger skin, processed in a similar manner, was used after cryopreservation. For cryopreservation, the avulsed fingertip skin was placed into chilled medium containing 10 vol% glycerol (1.37 M) and cooled in a −80 °C freezer at a cooling rate of approximately −1 °C/min. Immediately before use, the skin was thawed using the rapid warming method in a water bath (37 °C), followed by rinsing in physiological saline for at least 30 min.

### Case 1

**Figure 1.**
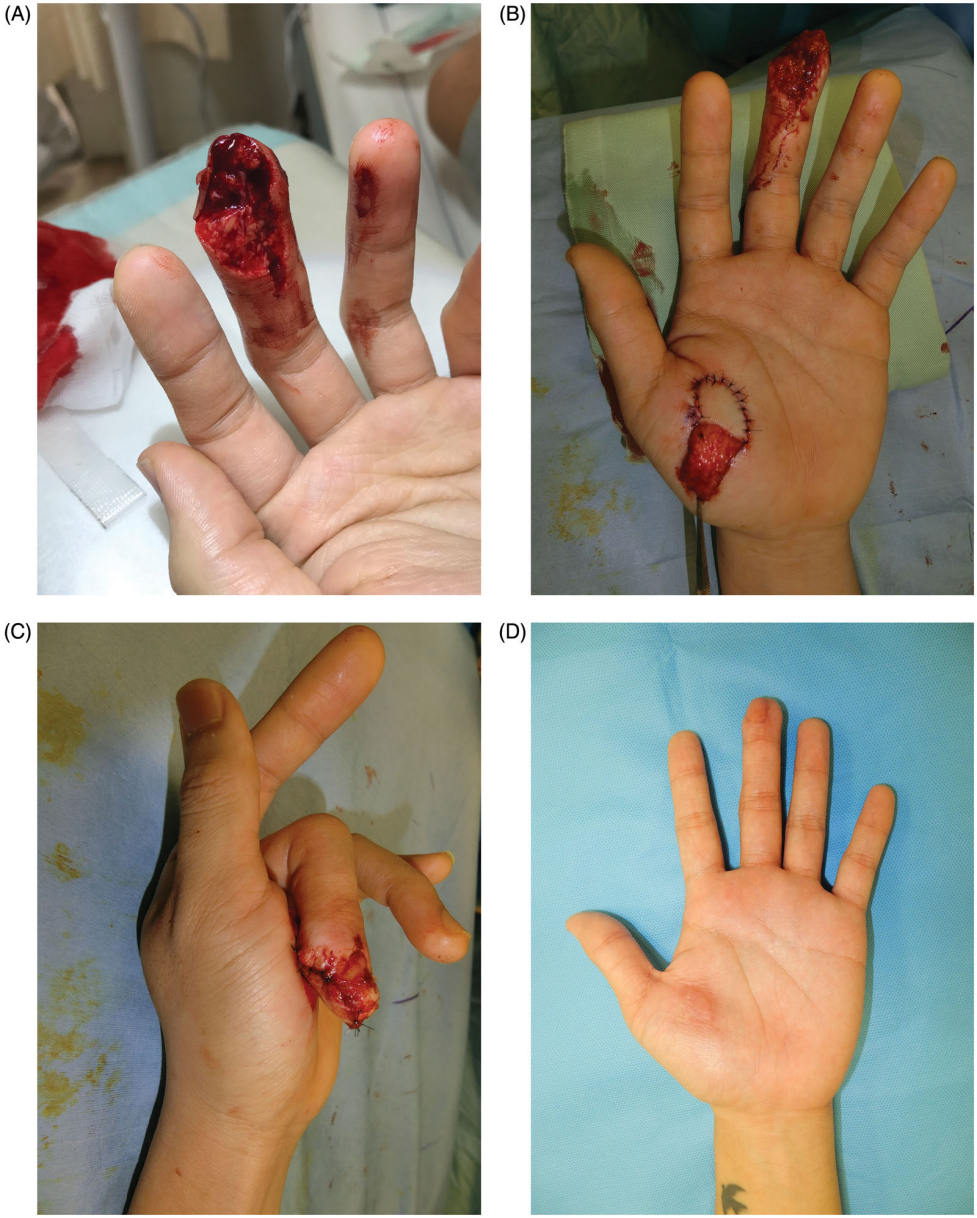
(A) Oblique avulsion of the left middle finger pad. The subcutaneous tissue of the avulsed finger had been intensely crushed, making replantation difficult. (B) Avulsed finger skin grafting to the thenar flap raised site. (C) Suturing of the flap without tension. (D) At 3 months after surgery, there is favorable fingertip form, no contracture in the skin grafted area, and good color/texture match.

A 23-year-old man was caught in a noodle machine, resulting in complete avulsion of the left middle finger (Tamai zone 2). Avulsion occurred obliquely along the finger pad (Figure 1(A)). Given the intense crush and exposed distal phalanx, replantation and immediate reconstruction were difficult to perform. Secondary reconstruction was planned, and the initial operation was completed by attaching the artificial dermis to the stump. The crushed finger skin after avulsion was cryopreserved. Nine days after injury, fingertip reconstruction was carried out by thenar flap under local anesthesia. The flap donor site was grafted the cryopreserved skin (Figure 1(B,C)). Two weeks later, the flap was detached. Both the graft and flap showed complete survival. The cryopreserved skin grafted matched well to the recipient site in terms of color and texture, and the reconstructed fingertip form was favorable (Figure 1(D)). No dysfunction was noted postoperatively.

### Case 2

**Figure 2.**
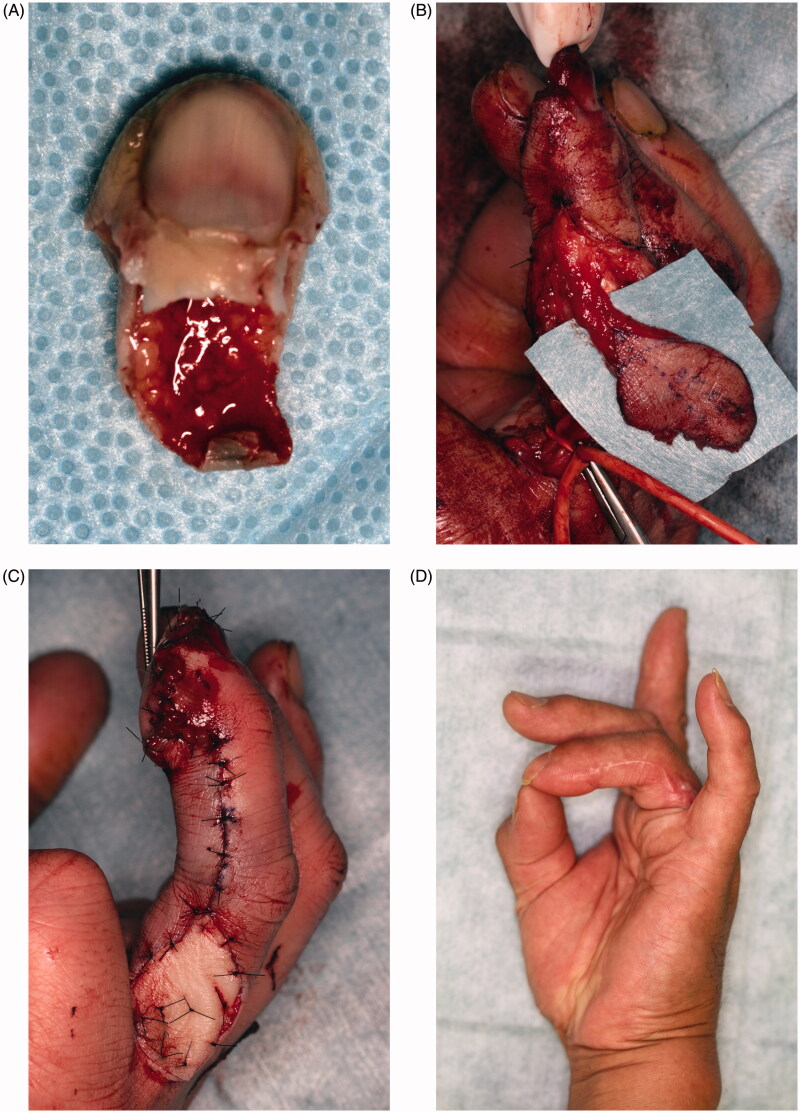
(A) Avulsed left ring finger tip. Intensely crushed. (B) Raising of a reverse digital artery flap. (C) Flap applied to the fingertip, with the avulsed finger skin grafted to the donor site. (D) At 6 months after surgery, there is favorable fingertip form and no contracture.

A 56-year-old man was caught in the soba noodle cutting machine, resulting in complete avulsion of the left ring finger (Tamai zone 1). Avulsion occurred obliquely along the finger pad (Figure 2(A)). Immediate reconstruction with a reverse digital artery flap from the ulnar side was conducted for the following reasons (Figure 2(B)): (1) the avulsed finger had been crushed intensely, making microscopic replantation difficult; (2) distal phalanx exposure; and (3) required large volume finger pad reconstruction. Full-thickness skin graft donated from the avulsed finger was implanted into the flap donor site (Figure 2(C)). Both the flap and donor site graft survived completely, yielding an outcome satisfactory both functionally and esthetically at 6 months postoperatively (Figure 2(D)).

### Case 3

**Figure 3.**
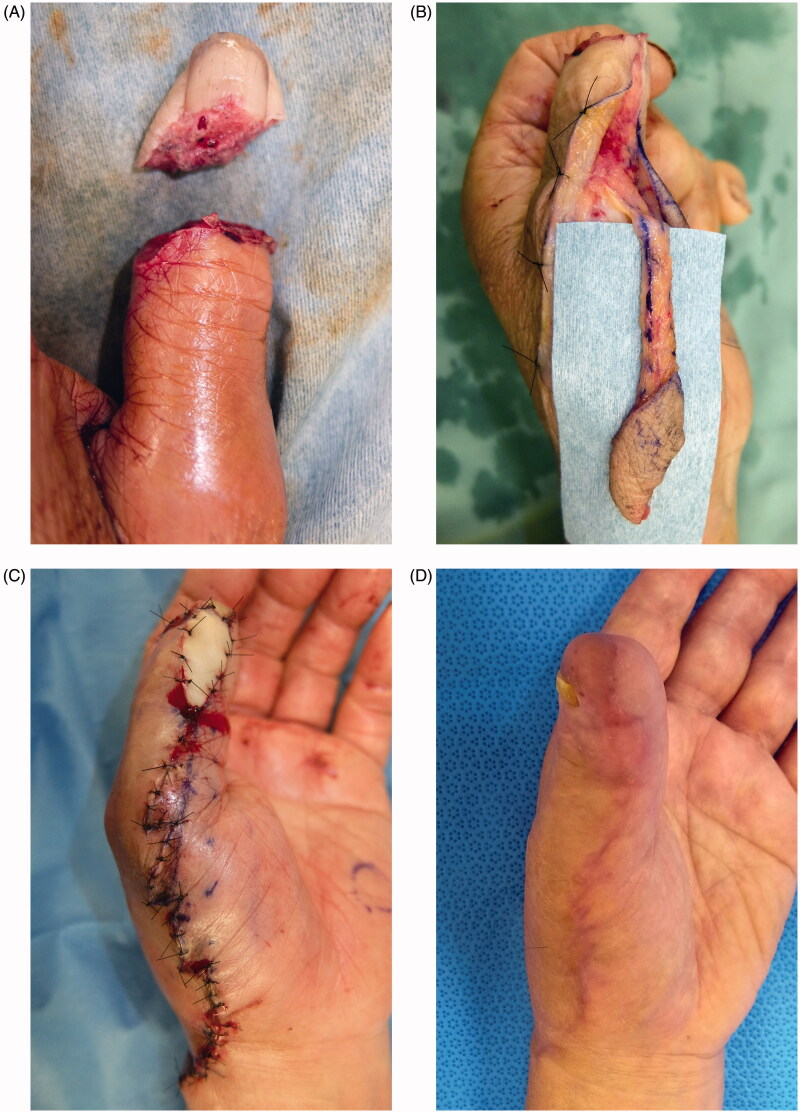
(A) Complete left thumb amputation. No vessel enabling anastomosis was available. (B) Flap of a reverse homodigital dorsal radial flap. (C) Flap grafting. Simple plication of the flap pedicle was difficult. Thus, the avulsed finger skin was grafted to the pedicle-everted area. (D) One month after surgery. No contracture of the donor site or no disturbance in motor function.

An 80-year-old man was injured with an electric saw, resulting in complete avulsion of the left thumb (Tamai zone 1). Avulsion occurred obliquely along the finger pad (Figure 3(A)). The avulsed finger had been intensely crushed, and it contained no artery or vein enabling microscopic anastomosis. Thus, we performed reconstruction with a reverse homodigital dorsal radial flap, which was designed on the dorsum pedis in a manner encompassing the dorsal radial digital artery (Figure 3(B)). It was raised to ensure 180-degree flap transposition to the thumb pad, covering the exposed bone. In this way, the tissue defect was filled. The full-thickness skin graft, donated from the avulsed finger, was applied to the raw surface partially formed on the vascular pedicle (Figure 3(C)). The flap and donor site graft survived completely without dysfunction at 1 month postoperatively (Figure 3(D)).

### Case 4

**Figure 4.**
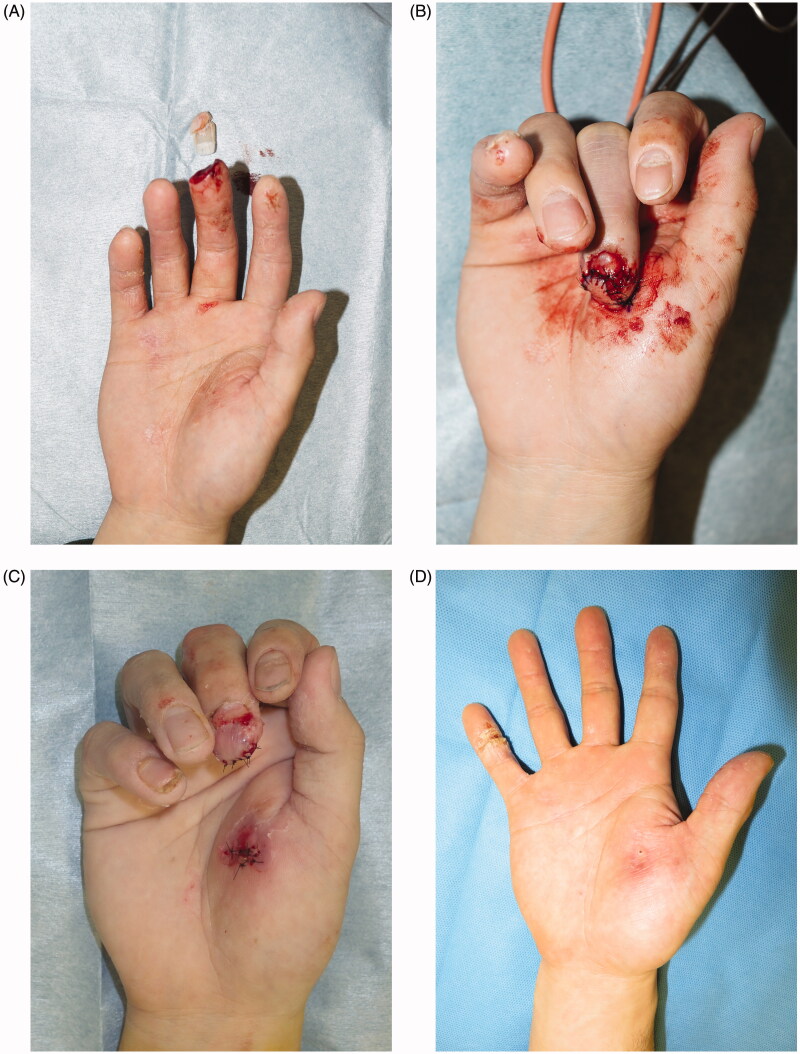
(A) Complete right middle finger tip amputation. (B) Fixed skin flap from the thenar site. (C) Detached the thenar flap after 2 weeks after first operation. (D) At 3 months after surgery, there is favorable fingertip form, no contracture in the skin grafted area, and good color/texture match.

A 22-year-old man was caught between the cart and floor, resulting in complete avulsion of the right middle finger (Tamai zone 1) (Figure 4(A)). Avulsion occurred obliquely along the finger pad. The subcutaneous tissue attached to the avulsed finger was scarce, and vessel exploration for anastomosis was difficult. Thus, reconstruction with a thenar flap was performed (Figure 4(B)). The full-thickness skin graft, donated from the avulsed finger, was applied to the donor site. The flap was detached 2 weeks postoperatively (Figure 4(C)). Both the graft and flap showed complete survival. The skin graft matched well to the recipient site in terms of color and texture, with favorable reconstructed fingertip form (Figure 4(D)). No dysfunction was noted postoperatively.

## Discussion

The goals of fingertip amputation treatments should include pain minimization, healing time optimization, sensibility and length preservation, painful neuroma prevention, nail deformity avoidance or limitation, minimizing time lost from work, and acceptable cosmetic appearance provision [1]. Fingertip avulsion treatment, including replantation or use of a composite graft or skin graft, reconstruction with a local flap if tissue replantation is judged difficult, and stump plasty, is conducted with these goals in mind. In cases rated as difficult for replantation, the fingertip is reconstructed often with a composite graft or skin graft or with a primary or secondary local flap procedure. Survival rate for the composite graft has been reported to be approximately 50% or less (varying depending on avulsion level or extent), and the size not exceeding 1 cm has been recommended. Thus, when treatment with the composite graft is selected, the treatment plan needs to be devised, taking into consideration prolongation of the duration of injury, possible need for additional surgery upon appearance of partial necrosis, and possible loss of avulsed finger skin [2,3]. For fingertip reconstruction with a local flap, the homodigital dorsal skin flap, reverse digital artery flap, and thenar flap are generally used. However, most operative procedures necessitate skin grafting into the donor site in both primary and secondary reconstructions. The skin graft utilizing the avulsed finger skin avoids sacrificing of the donor site, and the finger skin has very favorable texture and color match to the flap donor site.

In cases where replantation is not performed, the avulsed finger tends to be discarded. We have an impression that the avulsed finger, carried with the patient upon arrival, often retains its form as a skin even when the subcutaneous tissue has been intensely crushed. In all cases managed at our facility, the avulsed finger skin survived successfully. If the form has been preserved, the avulsed skin may be used for grafting. Therefore, this procedure seems to be useful in saving time and cost. This method is also applicable at facilities where a microscope is not available for use or manpower is insufficient. Considering that finger amputation often occurs in physical workers and early resumption of social activity is desirable for such workers, this method is expected to contribute to shortening the illness duration. We report only four cases of this procedure, therefore further study is needed to compare the functional and physiological results with the conventional methods of local flap reconstruction.

Bravo et al. reported that the viability of cryopreserved skin was approximately 60%, and that when stored at 4 °C, the viability was approximately 60% and 30% at days 1 and 7, respectively, post-preservation [4]. If the time until reconstruction is taken into consideration, it is desirable to cryopreserve the skin in cases where it is not used on the same day or reconstruction is planned later. Treatment utilizing the cryopreserved skin is primarily performed for burns using cadaver skin [5,6], but several reports in degloving injury are available [7,8]. According to our literature search, no report has been published concerning utilization of such skin as a graft for the fingertip reconstruction donor site. This procedure resulted in very favorable skin graft survival, even in cases where the avulsed finger skin was used for secondary reconstruction after cryopreservation.

## Conclusion

Our technique can avoid sacrificing of a new donor site through effective skin utilization. It is satisfactory in terms of shortening the treatment period/time and esthetic features. This approach seems to be useful as a new treatment option.
